# An Ultra-Low-Power RFID/NFC Frontend IC Using 0.18 μm CMOS Technology for Passive Tag Applications

**DOI:** 10.3390/s18051452

**Published:** 2018-05-07

**Authors:** Mayukh Bhattacharyya, Waldemar Gruenwald, Dirk Jansen, Leonhard Reindl, Jasmin Aghassi-Hagmann

**Affiliations:** 1 Institute for Applied Research, University of Applied Sciences Offenburg, 77652 Offenburg, Germany; waldemar.gruenwald@gmx.de (W.G.); info@dirk-jansen.de (D.J.); jasmin.aghassi-hagmann@hs-offenburg.de (J.A.-H.); 2 Department of Microsystems Engineering, University of Freiburg, 79098 Freiburg, Germany; reindl@imtek.uni-freiburg.de; 3 Institute of Nanotechnology, Karlsruhe Institute of Technology (KIT), Hermann-vom-Helmholtz-Platz 1, 76344 Eggenstein-Leopoldshafen, Germany

**Keywords:** RFID (radio frequency identification), NFC (near field communication), passive tag, comparator, demodulation

## Abstract

Battery-less passive sensor tags based on RFID or NFC technology have achieved much popularity in recent times. Passive tags are widely used for various applications like inventory control or in biotelemetry. In this paper, we present a new RFID/NFC frontend IC (integrated circuit) for 13.56 MHz passive tag applications. The design of the frontend IC is compatible with the standard ISO 15693/NFC 5. The paper discusses the analog design part in details with a brief overview of the digital interface and some of the critical measured parameters. A novel approach is adopted for the demodulator design, to demodulate the 10% ASK (amplitude shift keying) signal. The demodulator circuit consists of a comparator designed with a preset offset voltage. The comparator circuit design is discussed in detail. The power consumption of the bandgap reference circuit is used as the load for the envelope detection of the ASK modulated signal. The sub-threshold operation and low-supply-voltage are used extensively in the analog design—to keep the power consumption low. The IC was fabricated using 0.18 μm CMOS technology in a die area of 1.5 mm × 1.5 mm and an effective area of 0.7 $mm^{2}$. The minimum supply voltage desired is 1.2 V, for which the total power consumption is 107 μW. The analog part of the design consumes only 36 μW, which is low in comparison to other contemporary passive tags ICs. Eventually, a passive tag is developed using the frontend IC, a microcontroller, a temperature and a pressure sensor. A smart NFC device is used to readout the sensor data from the tag employing an Android-based application software. The measurement results demonstrate the full passive operational capability. The IC is suitable for low-power and low-cost industrial or biomedical battery-less sensor applications. A figure-of-merit (FOM) is proposed in this paper which is taken as a reference for comparison with other related state-of-the-art researches.

## 1. Introduction

RFID (radio frequency identification) technology has been widely employed for the design of the remotely powered telemetry systems since the 1950s. The RFID technology was first patented in the year 1973 [1], and since then it became more and more popular over the succeeding decades. How the RFID technology evolved over the years is well elaborated in [2]. The typical operating frequency of RFID varies from LF (low-frequency) range—100 kHz, HF (high-frequency) range—13.56 MHz to UHF (ultra-high-frequency) range—860–960MHz or 2.45GHz–5.7GHz.

From 2000 onwards, based on the existing RFID standards, a new set of communication protocols known as NFC or near field communication was introduced. Unlike RFID, NFC uses only the frequency range of 13.56MHz, and practically it is only functional over a distance less than 5cm [3,4]. Also, NFC enables a peer-to-peer (P2P) communication between a smart device and an NFC capable tag which is not possible with the RFID technology. Initially, NFC was introduced as an alternative to the existing Bluetooth standard, having a much shorter range and moderate data rate (maximum 424kbps). Unlike Bluetooth, NFC tags can be battery-less or passive.

There are five distinct kinds of applicable standards for NFC which are type 1&2-ISO/IEC 14443 A [5,6], type 3-JIS X 6319-4 (Felica) [7], type 4-ISO/IEC 14443 A/B [8] and type 5-ISO/IEC 15693 (18000-3) [9] as shown in Table 1. The proposed frontend IC is designed based on the ISO/IEC 15693 (18000-3) which corresponds to the Type-5 NFC tags. A commercial ISO 15693 RFID reader or an NFC capable smart device can be employed to communicate with the tag designed with the proposed IC.

RFID based telemetry systems have very broad application fields. Some of the recent examples of RFID based sensing applications are - measurement of concrete chloride ion concentration [10], inkjet printed passive RFID tag integrated with organic photodetectors [11], batteryless smart tag for orientation monitoring [12], humidity sensor for passive RFID applications [13], and indoor localization system [14,15]. Likewise, several biotelemetry application examples employing RFID are a wirelessly powered smart contact lens [16], miniaturized blood pressure telemetry system [17], continuous glucose monitoring [18], semi-passive implant for vital parameter monitoring in small animals [19], implantable blood flow sensor microsystem for vascular grafts [20], and continuous health monitoring system [21].

The RFID based contactless payment ought to be the future of the NFC technology [22,23,24]. Nevertheless, the application area for the NFC has also got broadened and involves diverse applications, for example, classroom access control [25] or IP based access to a sensor tag [26]. In recent years NFC technology has acknowledged its presence in biomedical applications too. Some of the examples in recent years are - dual carrier NFC based WPT (wireless power transfer) meant for small sized biomedical sensor applications [27], intraocular pressure measurement for monitoring glaucoma [28], wearable healthcare system including an ECG (electrocardiograph) processor and Instantaneous Heart Rate (IHR) monitor [29], and wireless fluorimeter for fully implantable biosensing applications [30].

Since the turn of the millennia, many state-of-the-art RFID or NFC protocol tag ICs were introduced. In this perspective, one of the earliest contributions was illustrated in [31]. The CMOS transceiver [31], compatible with the standard ISO 14443, was realized in a 0.5µm CMOS technology with an area of an area of $2\;mm^{2}$ and a power consumption of 5.3mW. With the scaling down of the CMOS technology, over the years, the overall power consumption and the IC area have been reduced drastically. For example, the frontend IC presented in [32] used FSK (frequency shift keying) demodulation, had a power consumption of 960µW and an area of $0.32\;mm^{2}$. A few years later, a passive tag IC compatible with the ISO-14443 type-B standard was presented in [33], which had an IC area of $1.1\;mm^{2}$ and a total power consumption of 360µW. More recently, the NFC tag IC proposed in [3] had a moderate analog power consumption of 67.7µW and an IC die area of $0.68\;mm^{2}$. The demodulation of 10% ASK modulated signal is always challenging in the presence of noise or jitter either from the environment or the from the internal circuits of the IC [3,33]. Different methods can be used to demodulate 10% ASK signal which is discussed in [33,34,35]. Most of the demodulator circuit consists of complex circuitries like unity gain buffers, high gain amplifiers, and comparators. An adaptive threshold voltage for the comparator to demodulate the 10% ASK signal, had been proposed in [3].

In accord with the recent development trend, the following key features are included in the state-of-the-art frontend IC presented in this paper:

- First and foremost, priority is given to low power consumption and smaller IC area which are the two key parameters for low-power, low-cost electronics.
- The digital interface and the RF transceiver are designed in accordance with the ISO/IEC 15693/NFC5 standard.
- As already discussed, the demodulation of the 10% ASK signal is challenging. An innovative and inexpensive method of ASK demodulation, comprising a comparator with a preset offset, is used for the design. The theoretical analysis of the comparator offset voltage depending on the operating region is discussed in detail in the paper. The primary advantage of this method is its simplicity, as it involves no complex circuitry.
- For the envelope detection of the ASK modulated signal, most of the designs use a resistor as a load, whereas for this design the power consumption of the bandgap reference circuit is used as the load. For this, it is essential that the bandgap reference circuit is stable and can operate with the substantial power supply noise. This approach helps to avoid an extra component like a resistor to be used exclusively by the envelope detector circuit, hence reducing the IC area and the power consumption.
- At the architectural level, multiple power supplies and sub-threshold region operations are used wherever possible to keep the power consumption low.
- A figure-of-merit (FOM) proposed in this paper is used for the comparison with other related works.

The proposed IC can be used to develop low-power industrial or biomedical passive sensor applications. Consequently, a passive tag is developed using the frontend IC, a microcontroller, a temperature and a pressure sensor. A smart device having an Android-based application software is used to readout the sensor data from the tag.

### Organization of the Paper

An overall system model and architecture is introduced in Section 2. Section 3 gives an overview of the low power design techniques used for the analog design. Section 4 discusses in detail the analog design. An overview of the digital design is provided in Section 5. Lastly, in Section 6, the measurement results of the design are presented and Section 7 substantiates the overall conclusion of the work.

## 2. System Model and Architecture

A typical RFID/NFC system consists of a reader device and a tag consisting of a frontend IC and a microcontroller as described in [36]. The voltage is induced by the reader to the tag via inductive coupling. The induced voltage on the tag side is dependent on the factors like the type of reader device used, antenna geometry, the inductance of the tag antenna, the distance between the reader device and the tag antenna and the coupling factor (k) in between the same [36].

The equivalent circuit models was elaborated in chapter 2 - [37] and chapter 2, 3 - [38], based on reader-tag system. The works [39,40,41] had employed an efficient antenna design model using PEEC (Partial Element Equivalent Circuit) method. The article [42] had introduced a mixed-resonant coupling model for efficient wireless power transfer.

**Figure 1:**
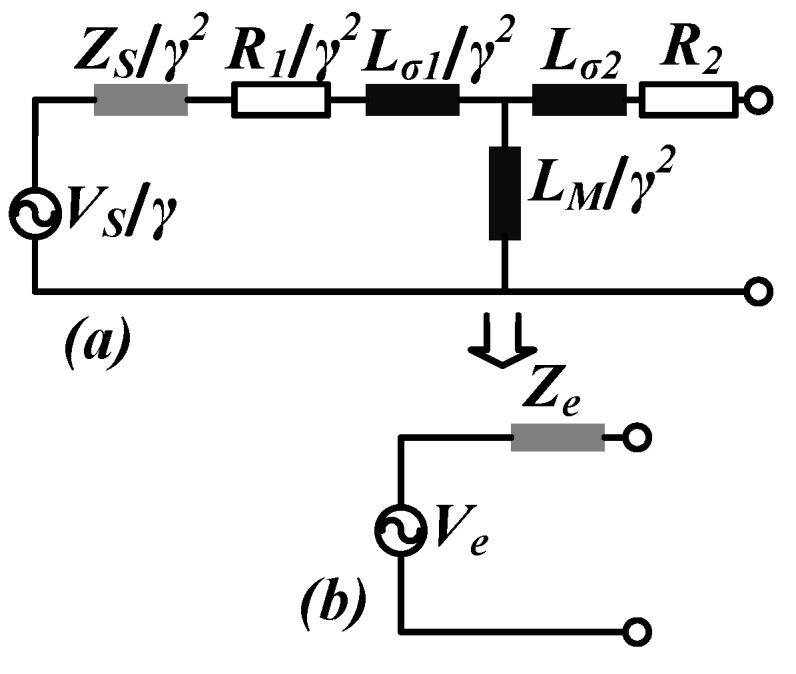
(**a**) Equivalent circuit representation used to derive the two terminal network after transforming the primary components to the secondary; (**b**) Thévenin’s equivalent circuit representation of the transformed circuit components [43].

In this case, for the power transfer, we are concerned with the voltage available for the tag in a resonance condition. A Thévenin’s equivalent model is adopted as a single network system for the simulation, after source transformation from the reader to the tag side [43]. Figure 1 indicates the equivalent circuit model after the transformation of the primary components—reader to the secondary—tag side.

In the equivalent model, $V_{S}$ represents the input voltage source; $Z_{S}$ represents the matched source impedance; $R_{1}$ and $R_{2}$ represent the winding resistances of the primary and secondary respectively; $L_{\sigma 1}$ and $L_{\sigma 2}$ are the primary and secondary leakage inductances; $L_{M}$ is the magnetizing reactance; γ indicates the primary to secondary turns ratio. Now $L_{M}$, $L_{\sigma 1}$ and $L_{\sigma 2}$ are expressed in terms of the total inductance of the antenna $L_{1}$ (reader antenna inductance) or $L_{2}$ (tag antenna inductance), coupling factor $k_{12}$ and γ which is detailed further in [43]. As per Thévenin’s theorem, the expression for the equivalent voltage $\left(V_{e}\right)$ for the open circuit condition is given as:

(1) $$V_{e}=\frac{1}{1+\frac{Z_{S}+R_{1}}{j\omega L_{1}}}\times \frac{k_{12}}{\gamma }\times V_{S}.$$

The series impedance ($Z_{e}$) is obtained from the ratio of equivalent open circuit voltage and short circuit current $\left(I_{sc}\right)$:

(2) $$I_{sc}=\frac{V_{S}}{\left(1+\frac{R_{2}+j\omega L_{\sigma 2}}{j\omega \times \left(\frac{L_{M}}{\gamma^{2}}\right)}\right)\left(\frac{Z_{S}}{\gamma^{2}}+\frac{R_{1}}{\gamma^{2}}+j\omega \frac{L_{\sigma 1}}{\gamma^{2}}\right)+R_{2}+j\omega L_{\sigma 2}}.$$

Hence the series impedance $Z_{e}$ is given as:

(3) $$Z_{e}=R_{2}+j\omega L_{2}-\left(\frac{k_{12}^{2}}{\gamma^{2}}\times \frac{1}{1+\frac{Z_{S}+R_{1}}{j\omega L_{1}}}\times j\omega L_{1}\right)$$

The equivalent model is discussed in detail in [43] and an experimental proof of the model is also provided, hence any further discussion is avoided here.

**Figure 2:**
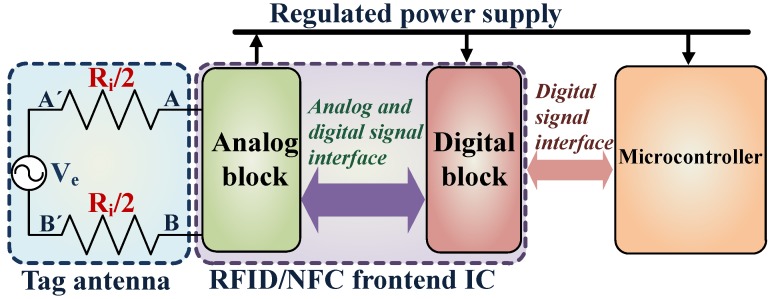
The basic system architecture of a passive tag, including the frontend IC and a microcontroller is presented here. The tag antenna is exemplified by the voltage source $V_{e}$ and the series resistance $R_{i}$.

The tag can be considered as an R (resistance)-L (inductance)-C (capacitance) parallel resonance circuit. When both the inductive and the capacitive reactance on the tag side are equal, resonance occurs. For resonance condition, the impedance of the circuit is maximized and purely resistive in nature, due to which the current and voltage are in phase. Hence at resonance, $Z_{e}$ is purely resistive and is represented as equivalent series resistance $R_{i}$. Figure 2, represents the complete system architecture of the tag along with the frontend IC, microcontroller, and the equivalent circuit representation of the tag antenna. The parameter values of $V_{e}$ and $R_{i}$ are listed in Table 2 for no load condition (open circuit) which are obtained by using the Equations (1) and (3) through numerical analysis and simulations [43]. The values of the $\left(V_{e-pk}\right)$ provided in Table 2 are adjusted according to the maximum allowed voltage level for the technology used which is 3.6V. The operation status on the extreme right-hand side column in Table 2 indicates the functional status of the tag.

## 3. Low Power Design Techniques Used for the Analog Design

Low power design techniques are essential to reduce the overall power consumption of the modern day electronic system. In a passive tag system, low-power consumption translates to the fact that it can operate in the presence of moderate field strength. Modern day designs incorporate several methods to reduce the overall power consumption of an electronic system. The tutorial presented in [44], lends a detailed insight into the world of ultra-low power VLSI (very-large-scale integration) circuit design. The paper [44] analyzes in extensive detail the low power design issues and approaches required. The work [45] discusses a trade-off in between precision, speed, topology, technology, and the task. From all these discussions, following approaches are adopted for the analog design:

- Weak inversion or sub-threshold operation is used wherever possible to keep the power consumption low. The sub-threshold drain current $I_{DSub-th}$ can be given as:

(4) $$I_{DSub-th}=I_{S}\frac{W}{L}e^{\frac{\left(V_{GS}-V_{th}\right)}{n\times v_{t}}}\left(1-e^{-\frac{V_{DS}}{v_{t}}}\right),$$

the term $I_{S}$ is the technology dependent sub-threshold current obtained for $V_{GS}$ = $V_{th}$, where $V_{GS}$ is the gate-source voltage and $V_{th}$ is the threshold voltage, $v_{t}$ is the thermal voltage, W/L is the aspect ratio and *n* is the sub-threshold factor [46]. In case $V_{GS}$ = $V_{th}$, Equation (4) can be further reduced to:

(5) $$I_{DSub-th}\approx I_{S}\frac{W}{L}e^{\frac{V_{GS}}{n\times v_{t}}}$$

For a constant W/L and $V_{GS}$, $I_{DSub-th}$ is exponentially dependent on $v_{t}$, having much smaller value in comparison to the drain current in saturation or strong inversion region. The sub-threshold operation of a transistor is illustrated in [46,47,48,49].

- Another method to reduce the overall power consumption is to use multiple power supply. Some of the analog circuits are driven by fixed supply voltage of $V_{DDI}$ = 1.2V, which is discussed later in Section 4.

These two approaches used for the design of the analog block is essential to keep the overall power consumption low.

## 4. Analog Block

**Figure 3:**
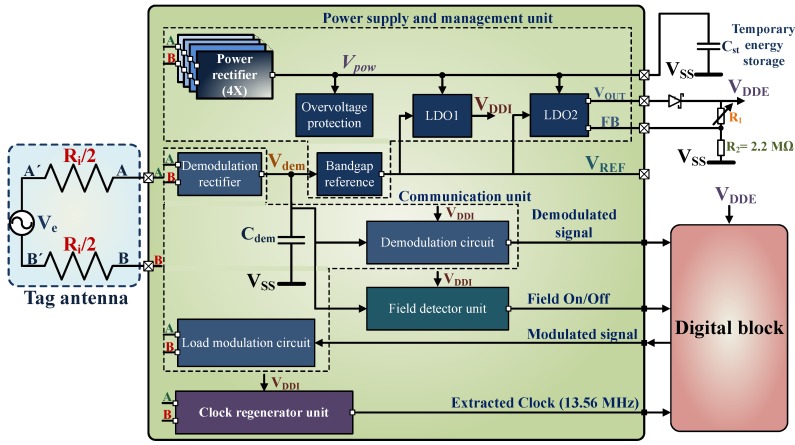
The system architecture of the analog block design.

The design architecture of the analog block of the IC is shown in Figure 3. On the basis of functionality, the analog block design can be divided into four sections which are:

1. Power supply and management unit
2. Communication unit
3. Field detector unit
4. Clock regenerator.

### 4.1. Power Supply and Management

The tag only uses the induced energy available from the RF (Radio Frequency) field of the reader device. Several RF-powering circuits have been proposed in [32,50,51,52,53]. The basic architecture of the power supply and management unit is similar to the designs presented in [50,52]. The power supply and management unit comprise of the over-voltage protection circuit along with the power rectifier, demodulation rectifier (partially), bandgap reference and low-dropout regulators (LDO1 and LDO2). LDO1 provides a fixed supply voltage of $V_{DDI}$ = 1.2V which supplies the demodulation unit, field detector unit and the clock regenerator unit. The digital block of the IC and the external components are powered by LDO2 with the voltage $V_{DDE}$ = 1.2 to 2.1V.

#### 4.1.1. Over-Voltage Protection

The over-voltage protection circuit and the ESD (Electro Static Discharge) diodes protect the IC from over-voltage conditions. Most of the RFID or NFC ICs use a bleeder circuit to dump the surplus energy to protect the internal circuits from getting damaged. The protection circuit can be implemented, by using stacked diodes connected in series with a resistor and a large shunting transistor [33]. As mentioned in [33], in case of an overvoltage condition, a shunting device is activated which in turn detunes the tag antenna which diminishes the input power to the rectifiers and also the effect of detuning is hard to predict. A simpler realization is by using a bleeder circuit consisting of stacked diodes in series with a resistance [3]. In this case, as the input power increases the effect of clamping will be strong, as a result, the modulation information will be lost due to the clipping effect. The bleeder circuit can also be utilized for the modulation purpose, as stated in [50], the modulation depth was compromised by the protection circuit, for which, a compensation was provided for this purpose. The circuit used in [34] has three different current paths to diminish the excess energy.

**Figure 4:**
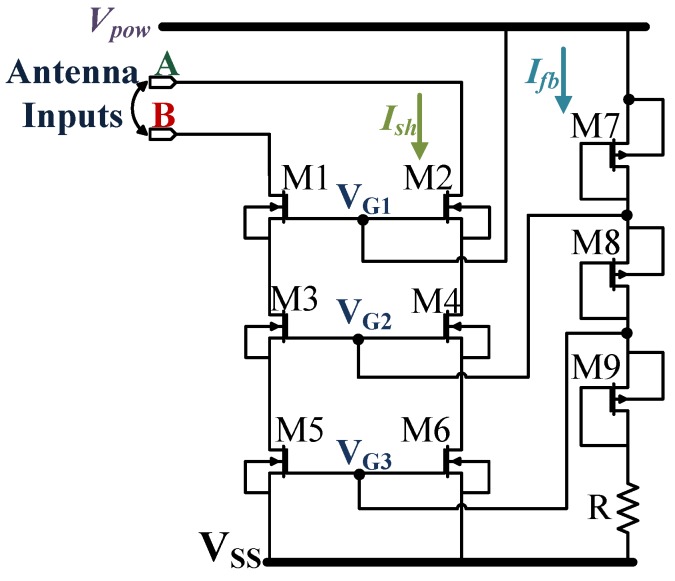
The over-voltage protection circuit. $V_{pow}$ is the output of the power rectifier.

For the proposed IC, the overvoltage protection circuit is implemented by using a bleeder circuit composed of transistors M7-9 and resistor R as shown in Figure 4. Instead of using only one pair of shunt devices, it uses three pairs of staggered NMOS transistors (M1-6). The voltage is reduced slowly to reduce the effect of hard clamping. When $V_{e-pk}$ is greater than the maximum allowed voltage which is 3.6V, the circuit becomes fully active. The individual gate voltages $V_{G1-3}$ are provided by the power rectifier output voltage $V_{pow}$, discussed later in the Section 4.1.2 and the bleeder circuit’s diode connected devices M7-9. The clamped transistors for each of the pairs are placed inside separate wells so that they carry only one-third of the input voltage. The transistor pairs are initially driven to the triode region, as the input voltage increases they are in saturation. The transistor pair M5-6 is made wider than the other two pairs to facilitate maximum current dumping by the shunt devices.

Under typical conditions, the maximum value of $I_{sh}$ (shunt current) is 3mA for a $V_{e-pk}$ of 3.6V, which corresponds to $V_{pow}$ of 2.5V. As the input voltage increases beyond the maximum allowed limit, the protection circuit disperses around 20mA in the absence of any external load. Thus, $V_{e-pk}$ is maintained within the maximum allowed voltage limit of 3.6V. The maximum value of $I_{sh}$ achieved by simulating the overvoltage condition for $V_{e-pk}$ of 3.6V and different load conditions using Table 2.

From the knowledge of $I_{sh-max}$ = 20mA and the maximum value of $V_{pow-max}$ = 2.5V, the minimum value of the total shunt resistance $\left(R_{sh}\right)$ obtained is 125. This $R_{sh}$ is divided among the channel resistances of the transistor pairs M1-2, M3-4 and M5-6 $\left(R_{ch\left(1-6\right)}\right)$ approximately in a ratio of 9:2:1. The value of the channel resistance for each of the stages is chosen by the device dimension $\left(\frac{W}{L}\right)$ and the respective gate voltages $V_{G(1-3)}$ which is given by:

(6) $$R_{ch(1-6)}=\frac{1}{\beta \times {\left(\frac{W}{L}\right)}_{\left(1-6\right)}\left(V_{GS(1-3)}-V_{th,n}\right)}$$

where β is the process factor, $V_{GS}$ is the gate source voltage and $V_{th,n}$ is the respective threshold voltages for the device M1-6. The gate voltages $V_{G(1-3)}$ are obtained from the voltage divider comprising transistors M7-9 and resistor R = 240kΩ. The devices M7-9 has equal channel resistances i.e., $R_{ch7}$ = $R_{ch8}$ = $R_{ch9}$ = 400kΩ for the $V_{pow-max}$ and the current through the bleeder circuit denoted as $I_{fb}$ (refer Figure 4) is 2µA.

(7) $$V_{G1}=V_{pow-max}$$

(8) $$V_{G2}=V_{pow-max}-I_{fb}\times R_{ch7}$$

(9) $$V_{G3}=V_{pow-max}-I_{fb}\times \left(R_{ch7}+R_{ch8}\right).$$

From Equations (7)–(9), the gate voltages are $V_{G1}$ ≈ 2.5V, $V_{G2}$ ≈ 1.9V and $V_{G3}$ ≈ 1.3V. The total area of the overvoltage protection circuit is $0.066\;mm^{2}$, which is 9% of the entire die area. The ESD protection for all the analog signals including the antenna inputs is provided by the analog pad cells. For this, 3.3 V (+10% maximum tolerance) analog ESD I/O (input-output) thick oxides pad cells are used. Each of the analog pad cells is capable of carrying a maximum of 60.8mA current at 100 °C, for a very short period of time, as provided by the foundry. The maximum potential used by the ESD protection circuit is that of the $V_{pow}$ and the minimum is that of the $V_{SS}$. The protection circuit dissipates the excess energy in a smooth manner, without the hard clamping of the modulated signal. Moreover, being a passive system, the fixed power consumption of the IC itself and the variable power consumption by the external load also plays a vital role, as they determine the operating limit of the protection circuit.

#### 4.1.2. Power Rectifier Circuit

**Figure 5:**
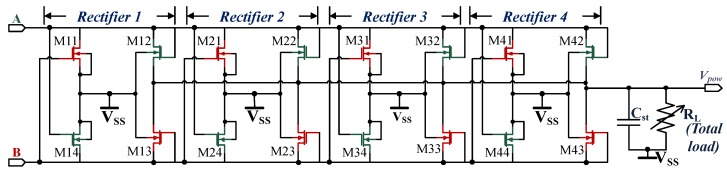
The circuit schematic of the four parallel NMOS cross-coupled rectifier.

The power rectifier circuit, as shown in Figure 5, provides power to the low drop out regulators—LDO1 and LDO2. It is possible to use either passive rectifiers [54,55] or active rectifiers [53,56]. The active rectifiers have higher power conversion efficiency compared to the passive rectifiers but require more circuit complexity. Active rectifiers are more useful for semi-passive applications, as the available energy can be used for long-term storage purpose, either by using a rechargeable battery or a double layer capacitor. The working condition is much more dynamic in the case of a passive system, which is only active when the RF field is on. The working range depends on the available energy from the field and also on the demodulation and the modulation signal. Again the available energy is dependent on the attached load, which affects the demodulation and the modulation signal. For example, there can be a scenario where the rectified voltage is sufficient but, still, the communication is impossible. This scenario can arise, in case either the reader or the tag is unable to detect the weak modulation signal sent to one another. A passive rectifier is a good trade-off for a batteryless tag application, as it involves neither complex circuit or any auxiliary components.

In this design, four parallel gate cross-connected NMOS rectifier units are used as shown in Figure 5. Where $R_{L}$ represents the total load supported by the rectifier and capacitor $C_{st}$ is used for temporary storage. The transistors Mn1 and Mn4 function as switches whereas Mn2 and Mn3 are the diode-connected devices, where n = 1 to 4. The phase-inverted antenna inputs A and B switch on each of the even and odd numbered transistor pairs in respective clock cycles. Each of the switches is turned on by the complete swing of the voltage $V_{e-pk}$ at respective clock cycles. For example voltage $V_{e-pk}$ at the input A creates a conducting path between input A, even numbered diode-connected devices (Mn2 and Mn4) and the load $R_{L}$. The devices Mn2 and Mn4 are turned on, which means input B has the same potential as VSS. At this point, Mn1 and Mn3 are switched off. In the next clock cycle, the same process is repeated where the odd-numbered diode connected devices are switched on and the even numbered are switched off. The cross-coupled NMOS rectifier is discussed in detail in the works [50,52], hence any further explanation is avoided here.

The rectifier unit consumes a power of 122pW and can support a maximum load of 8.5mW.

#### 4.1.3. Bandgap Reference

The bandgap reference voltage is generated, by adding the PTAT or proportional to absolute temperature and CTAT or complementary to absolute temperature voltages. For the first time, Widlar introduced the fundamental concept of the bandgap reference circuit [57,58]. The basic working principle of the bandgap reference circuit is explained in [59,60]. Only the key design parameters of the bandgap reference circuit are discussed in this section.

**Figure 6:**
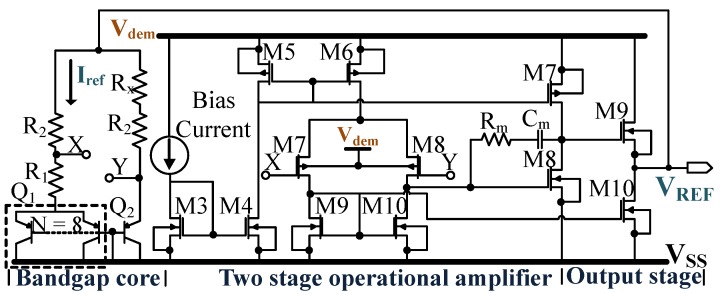
Bandgap reference circuit schematic.

The bandgap reference circuit used is shown in Figure 6, where $V_{REF}$ is the reference voltage generated. The demodulation rectifier powers the circuit by the rectified voltage $V_{dem}$ (refer Section 4.2). The power consumption of the bandgap reference circuit act as the load for the envelope detector circuit, which is discussed later in the Section 4.2. The reference circuit consists of a bandgap core generating the two temperature coefficients of PTAT and CTAT, a two-stage operational amplifier and a source follower as the output stage. The bandgap core consists of the BJTs $Q_{1}$—8 in parallel and $Q_{2}$—single BJT. The collector currents of $Q_{1}$ and $Q_{2}$ also endure temperature dependency. For this N+ Poly-resistors are used, having a smaller variation ±1% for −30 °C to 85 °C. The average value of $V_{REF}$ is 1.215V for the nominal temperature of 25 °C. The PNP BJT model $Q_{1}$ used has an emitter area of $10\times 10\;µm^{2}$ and a base area of $21.2\times 21.2\;µm^{2}$. The resistor $R_{x}$ provides partial curvature compensation for $V_{REF}$. The value for $R_{x}$ is obtained from the Monte-Carlo simulation for different process corners.

A two-stage op-amp operating in the sub-threshold region is used as an error amplifier to track the node voltages at X and Y (refer Figure 6). The output buffer stage is implemented using a source-follower configuration. The simulated loop gain of the op-amp is 112dB and has a GBW (gain-bandwidth) of 12.4MHz. The simulated PSR (power supply rejection) is approximately -52.42dB at 50kHz and -43dB at 100kHz. For improving the loop stability, a Miller compensation is provided in the form of $R_{m}$ = 100 KΩ and $C_{m}$ = 2.06 pF, as shown in Figure 6. The inclusion of the miller compensation degrades the PSR of the circuit at the higher frequency to an extent which can be compensated by including a small decoupling capacitor of 5pF at the output $V_{REF}$ of the reference circuit. The higher gain of the op-amp helps to increase the PSR at the lower frequency.

The minimum value of $V_{dem}$, required to turn on the circuit is 1.7V and the power consumed for the same is 5.1µW. The total layout area of the bandgap reference circuit is $0.044\;mm^{2}$. The supply voltage sensitivity is 152 µV/V at 25 °C for $V_{dem}$ ranging in between 1.7 V to 2.5 V. The simulation result reveals, the $V_{REF}$ has a variation of ±0.33%, over the temperature range of −30 °C to 85 °C with respect to the nominal value. The simulated startup time for the bandgap reference circuit is 5µs.

#### 4.1.4. Low Drop out Regulator Circuit (LDO2 & LDO1)

In low power applications, mostly single stage regulators are used, due to lower power consumption as mentioned in [61]. The fundamentals of the LDO are well presented in [61,62,63,64]. A comprehensive explanation of an LDO is illustrated by the authors in [65], in this paper only the critical parameters are presented.

**Figure 7:**
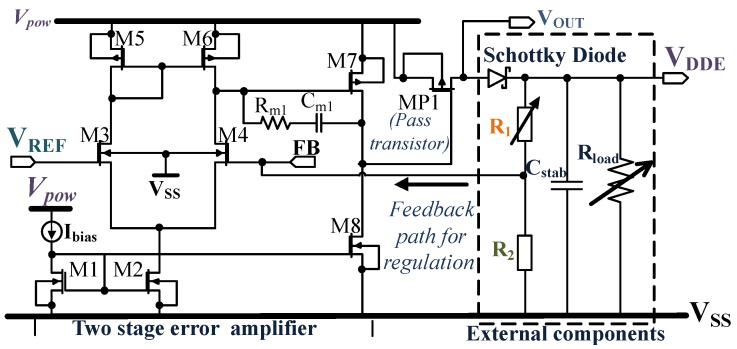
Schematic of LDO2 together with the external components.

Figure 7 shows LDO2 along with the external components which provide a voltage $V_{DDE}$ for all the external components such as the microcontroller, sensors and the digital core of the IC. The LDO2 presented here, consists of a two-stage error amplifier and a PMOS (MP1) as the pass transistor. The output voltage $V_{OUT}$ is set by $V_{REF}$, $R_{1}$ and $R_{2}$. The external Schottky diode prevents discharging of the capacitor $C_{stab}$ through the pass transistor. The Schottky diode is not needed if the pass transistor is implemented using an NMOS. But this will increase the overall dropout voltage by ≈0.6V. If the dropout voltage for the LDO2 is $V_{d2}$, and that of the Schottky diode is $V_{dS}$, the expression for $V_{OUT}$ and $V_{DDE}$ can be given as:

(10) $$V_{OUT}\approx V_{REF}\times \left(1+\frac{R_{1}}{R_{2}}\right)$$

(11) $$V_{DDE}=V_{OUT}-V_{dS}.$$

$R_{2}$ = 2.2MΩ is fixed and $R_{1}$ is chosen depending on the desired value of $V_{DDE}$ which can range from 1.2V to 2.1V. LDO2 has a line regulation of 4.23µV/V for the maximum load current of 4mA and a nominal dropout voltage of 150mV. The PMOS pass transistors act as an amplifier, which adds up a non-dominant pole. The stability depends on the load current, because of which the transconductance of the output pass transistor varies, which affects the non-dominant pole. The capacitor $C_{stab}$ = 1µF establishes stability for all possible load conditions. The quiescent current including the resistor $R_{2}$ is 3.6µA, which is very low for a two-stage LDO. The load regulation of the LDO2 varies from 354nV/mA to 683nV/mA for $V_{DDE}$ = 1.2V to 2.1V respectively.

The LDO1 supplies the internal components of the analog block as shown in Figure 3. It is similar to the LDO presented in Figure 7 except that it has a fixed output voltage VDDI = 1.2V and can support a maximum load current of 80µA.

### 4.2. Communication unit

The NFC communication block includes a demodulator and a modulator circuit.

#### 4.2.1. Demodulation and Envelope Detector Circuit

**Figure 8:**
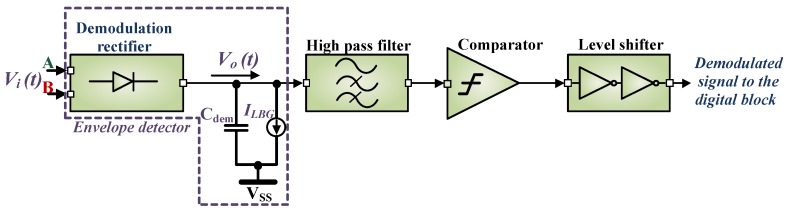
The complete demodulation circuit where $I_{LBG}$ represents the current consumption of the bandgap reference circuit.

Figure 8 shows the demodulation circuit which consists of the envelope detector, high pass filter, comparator and the level shifter. The envelope detector circuit is used to extract the information from the modulated signal sent by the reader to the tag. For the envelope detection, a resistive and a capacitive load is required to extract the message from the input signal. Most of the designs [3,31,33,34,50] presented in recent times use either resistor in silicon process or an active device controlled by a bias voltage as the load for the envelope detection. Every extra component requires more area and causes power dissipation. A novel approach is adopted to minimize the complexity and area, the bandgap reference circuit which dissipates a current $I_{LBG}$ is used as the demodulation load as shown in Figure 8. The demodulation rectifier is similar to the power rectifier unit, but with much smaller device dimensions as it needs to support a maximum of 85µW. So there is a trade-off in between the power dissipated and the area occupied by the resistive load. A higher value of resistance in the range of 500–600 kΩ will dissipate less power but requires a larger area. For example, if a separate load of 600kΩ is used for the envelope detector circuit, it dissipates a minimum of 5µW to a maximum of 36µW and occupies an area of $0.006\;mm^{2}$. This extra power dissipation and component area is avoided by using the bandgap reference circuit as the load for the envelope detector circuit.

The working principle of the gate cross-coupled NMOS rectifier has been discussed in Section 4.1.2. Each time the diodes are forward biased, $C_{dem}$ is charged up to the peak output voltage $V_{o}\left(t\right)$ of the demodulation rectifier. The relationship in between the charging time constant $\tau_{ch}$ and the period of the carrier $T_{ca}$ can be given as:

(12) $$\tau_{ch}\ll T_{ca}$$

where $\tau_{ch}$ = $R_{i}\times C_{dem}$ and $T_{ca}$ = $\frac{1}{f_{c}}$, $f_{c}$ is the carrier frequency (13.56 MHz). The discharging time constant $\left(\tau_{dis}\right)$ is large enough so that $C_{dem}$ discharges slowly through the load resistor $R_{dem}$ when the input peak voltage $\left(V_{i}\left(t\right)\right)$ drops for a short while due to the modulation. The $\tau_{dis}$ of the demodulation capacitor also depends on $\frac{dV_{i}\left(t\right)}{dt}$, the discharge rate of the capacitor is smaller than this. So the relationship among all the concerned time constants can be given as:

(13) $$T_{ca}\ll \tau_{dis}\ll T_{m}$$

where $\tau_{dis}$ = $R_{dem}\times C_{dem}$ and $T_{m}$= $\frac{1}{f_{m}}$, where $f_{m}$ indicates message bandwidth. The value of $R_{dem}$ reduces with the increase in the input voltage $V_{i}\left(t\right)$ but as obtained from Equation (13) it is important to maintain $\tau_{dis}$. In practice $R_{dem}$ is the active load of the bandgap reference circuit. As obtained from the simulation, $R_{dem}$ varies from 600kΩ to 260kΩ with power dissipations of 5µW to 36µW respectively. In practice $T_{ca}$ ≈ 74ns and $T_{m}$ = 6-13.94µs [66]. A capacitor of 5pF is used for $C_{dem}$, where the average minimum and maximum value of $\tau_{dis}$ is ∼1.3µs and ∼3µs respectively.

#### 4.2.2. Comparator with High Pass Filter and Level Shifter

In this section, we have discussed the design details of the comparator, including the technique used to introduce a forced offset voltage in the comparator circuit. At first, the operating conditions for the demodulator are discussed, followed by a detailed explanation of the offset voltage in the comparator design. The expression for the offset voltage depending on different operating regions, including mismatch conditions is discussed in detail.

The demodulator is designed to demodulate minimum of 10 % ASK modulated signal sent by the reader to the tag. Typically, the modulation amplitude ranges within ≈25mV to ≈400mV for this design, which depends on the field strength, coupling factor and the distance between the reader and the tag. Several methods can be used to demodulate 10% ASK signal which is discussed in [33,34,35]. In [33,34], the demodulator circuit consists of two unity gain buffers, a high gain amplifier, and a hysteresis comparator. The work [3] uses an adaptive threshold voltage for the comparator to demodulate the 10% ASK signal. Most of the cited work uses a comparator and some additional circuitries for the demodulation. Thus, the comparator has a stable reference for comparison.

**Figure 9:**
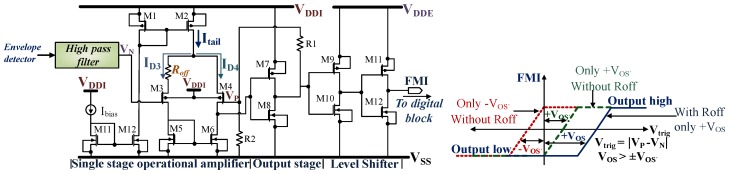
Demodulation comparator circuit along with the output (FMI) characteristics.

Figure 9 shows the closed loop comparator used for this design along with the high-pass filter, input nodes $V_{N}$ and $V_{P}$, and the output signal FMI (Field Modulation In). The high-pass filter has a cutoff frequency of 11.9kHz, which strips the modulation signal from the envelope signal obtained from the demodulation rectifier. The input offset voltage of the comparator influences the trigger point $V_{trig}$ for the transition of the comparator output from high to low and vice versa. $V_{trig}$ can be defined as:

(14) $$V_{trig}=\left|V_{P}-V_{N}\right|.$$

In the absence of any offset, $V_{trig}$ only depends on the hysteresis voltage $\left|V_{hys}\right|$. If the input signal is greater than $V_{trig}$ the output will go high and vice versa. In the presence of offset voltage $\left|V_{OS^{\prime }}\right|$, $V_{trig}$ will be shifted accordingly.

Primarily the offset voltage can be classified as the systematic and random offset. The systematic offset voltage can be estimated via simulations for various operating conditions like temperature, process variations, and tolerances. The random offset occurs because of random spreading of different parameters. The principal source of the mismatch for the differential pair M3-4 is the variation in the channel resistance $R_{ch,5-6}$ of the load devices M5 and M6. Due to mismatches, channel resistances will vary by a small value of $\Delta R_{ch}$ ≈ 1% as obtained from the simulation. Additionally, the random variation in the threshold voltages $V_{th}$ and the device dimensions (W/L) of the input devices will result in random mismatches which are hard to anticipate. The systematic offset can be reduced by proper layout techniques or by employing dynamic offset cancellation techniques. In general, large devices are used to avoid the effect of mismatches which in turn reduces the systematic offset voltage. Besides, the total gate area is essential for scaling down the effect of mismatches, as merely the layout style doesn’t make much difference [67]. The total offset voltage is the sum of the systematic and random offset voltage. It is plausible to reduce the offset voltage for the differential pair, but it is arduous to get rid of it entirely.

Moreover, the result of various spreads and variations may result in $\pm V_{{OS}^{\prime }}$ and largely depends on the operating region of the devices which is discussed later. In absence of any kind of mismatch, the comparator has an offset voltage of 44µV in weak inversion region. For the same operating region, we performed the Monte-Carlo simulation with 200 samples and a 3σ standard deviation where $\pm V_{{OS}^{\prime }}$ = 7.5mV including all sources of mismatch. The Monte-Carlo simulation provides with the spread of the offset voltage due to various mismatch conditions. In case the circuit has $-V_{{OS}^{\prime }}$, the default value of FMI will be high even in the absence of any demodulation signal. In case it has $+V_{{OS}^{\prime }}$ < 10mV, small noise or jitter may cause a change of state for the comparator. Both these conditions will result in an error in the demodulation process.

Consequently, an additional variation in the tail current is introduced in the form of $R_{off}$ as shown in Figure 9, which provides an extra positive offset voltage $V_{off}$. As $R_{off}$ >> $\Delta R_{ch}$ , it is the predominant contributor to the offset voltage. This result in a total offset voltage of $V_{OS}$ in the circuit, which shifts the reference point to $V_{trig}$ + $V_{OS}$ for all possible mismatches, $V_{OS}$ can be expressed as:

(15) $$V_{OS}=\pm V_{{OS}^{\prime }}+V_{off}.$$

The change in the drain current $I_{D}$ of M3 due to the addition of the series resistance $R_{off}$ is $I_{off}$, where $I_{off}$ ≪ $I_{D}$. The expression for the offset voltage can be obtained by considering the systematic mismatches i.e., the mismatches in the device dimensions, threshold voltage, and $\Delta R_{ch}$. Now the physical value of the offset voltage largely depends on the region of operation for the transistors M3-6 which depends on the input overdrive voltage.

Next, we theoretically analyze the effect of mismatch and process variations on the expression of $V_{OS}$. We obtain the expressions of $V_{OS}$ for both the strong inversion and the weak inversion regions. For the sake of simplicity of the analysis, the channel length modulation λ or the variation in the body effect coefficient γ is neglected.

Strong inversion operation:

The threshold voltage for M4 be $\left|V_{th}\right|$ and M3 is ($\left|V_{th}\right|+\left|{\Delta V}_{th}\right|$), aspect ratio for M4 and M3 is $\left(W/L\right)$ and $\left(W/L\right)+\left(\Delta W/L\right)$ respectively. The drain current $I_{D4}$ for M4 can be given as:

(16) $$I_{D4}=I_{D}$$

where $I_{D}=I_{tail}/2$, $I_{tail}$ is the tail current for the differential pair. Similarly for M3 it can be shown that the drain current $I_{D3}$ having a small variation of $\Delta I_{D}$ due to the load devices M5 and M6 can be given as:

(17) $$I_{D3}=I_{D}+\Delta I_{D}.$$

Now if we include the offset current $I_{off}$ in Equation (17), it can be re-written as:

(18) $$I_{D3}=I_{D}+\underset{I_{OS}}{\underset{︸}{\Delta I_{D}+I_{off}}}=I_{D}+I_{OS}$$

where $I_{OS}$ is the total offset. Similarly the respective aspect ratios for M4 and M3 can be expressed as:

(19) $${\left(\frac{W}{L}\right)}_{4}=\left(\frac{W}{L}\right)$$

(20) $${\left(\frac{W}{L}\right)}_{3}=\left(\frac{W}{L}\right)+\Delta \left(\frac{W}{L}\right).$$

Now the input offset voltage can be given as:

(21) $$V_{OSin}=\left|V_{GS4}\right|-\left|V_{GS3}\right|.$$

So the voltages $V_{GS3}$ and $V_{GS4}$ can be expressed in terms of device dimensions, drain current and process factor:

(22) $$V_{GS3,4}=\frac{2I_{D3,4}}{gm_{3,4}}+\left|V_{th3,4}\right|$$

where $I_{D3,4}$ indicates the drain current, $g_{m3,4}$ indicates the transconductance and $\left|V_{th3,4}\right|$ are the threshold voltages for the transistors M3 and M4. The transconductance can be expressed in terms of device dimensions, process factor and drain current:

(23) $$g_{m3,4}=\sqrt{2\mu_{p}C_{ox}{\left(\frac{W}{L}\right)}_{3,4}I_{D3,4}}$$

where ${µ}_{p}$ indicates the mobility of the holes for the PMOS devices, ${\left(\frac{W}{L}\right)}_{3,4}$ is the aspect ratio for transistors M3 and M4.

Replacing Equation (23) in Equation (22) one can express Equation (21) as:

(24) $$V_{OSin}=\sqrt{\frac{2I_{D4}}{\mu_{p}C_{ox}{\left(\frac{W}{L}\right)}_{4}}}+\left|V_{th4}\right|-\sqrt{\frac{2I_{D3}}{\mu_{p}C_{ox}{\left(\frac{W}{L}\right)}_{3}}}-\left|V_{th3}\right|.$$

Replacing $I_{D4}$ and $I_{D3}$ from the Equations (16) and (18) respectively as well as the aspect ratios for M4 and M3 from Equations (19) and (20) respectively, Equation (24) can be re-written as:

(25) $$V_{OSin}=\sqrt{\frac{2I_{D}}{\mu_{p}C_{ox}\left(\frac{W}{L}\right)}}\left[1-\sqrt{\frac{1+\frac{I_{OS}}{I_{D}}}{1+\frac{\Delta (\frac{W}{L})}{\left(\frac{W}{L}\right)}}}\right]+\left|V_{th4}\right|-\left|V_{th3}\right|.$$

As $\left|V_{th4}\right|$ =$\left|V_{th}\right|$ and $\left|V_{th3}\right|$= $\left|V_{th}\right|+\left|{\Delta V}_{th}\right|$, Equation (25) is given as:

(26) $$V_{OSin}=\sqrt{\frac{2I_{D}}{\mu_{p}C_{ox}\left(\frac{W}{L}\right)}}\left[1-\sqrt{\frac{1+\frac{I_{OS}}{I_{D}}}{1+\frac{\Delta (\frac{W}{L})}{\left(\frac{W}{L}\right)}}}\right]+\left|\Delta V_{th}\right|.$$

The terms $\frac{I_{OS}}{I_{D}}$ and $\frac{\Delta \left(\frac{W}{L}\right)}{\left(\frac{W}{L}\right)}\ll 1$, hence the term ${\left(\sqrt{1+\frac{\Delta (\frac{W}{L})}{\left(\frac{W}{L}\right)}}\right)}^{-1}$≈ $\left\{1-\frac{\Delta (\frac{W}{L})}{2\left(\frac{W}{L}\right)}\right\}$ and $\sqrt{1+\frac{I_{OS}}{I_{D}}}$ ≈ $\left\{1+\frac{I_{OS}}{2I_{D}}\right\}$ (binomial theorem), Equation (26) is given as:

(27) $$V_{OSin}=\sqrt{\frac{2I_{D}}{\mu_{p}C_{ox}\left(\frac{W}{L}\right)}}\left[1-\left\{1+\frac{I_{OS}}{2I_{D}}\right\}\times \left\{1-\frac{\Delta (\frac{W}{L})}{2\left(\frac{W}{L}\right)}\right\}\right]+\left|\Delta V_{th}\right|.$$

(28) $$\Rightarrow V_{OSin}=\frac{1}{2}\sqrt{\frac{2I_{D}}{\mu_{p}C_{ox}\left(\frac{W}{L}\right)}}\left[\frac{\Delta (\frac{W}{L})}{\left(\frac{W}{L}\right)}-\frac{I_{OS}}{I_{D}}\right]+\left|\Delta V_{th}\right|.$$

Equation (28) can be further expressed in terms of $R_{ch}$ and $R_{off}$ . In absence of any kind of mismatches:

(29) $$I_{D3}\times R_{ch3}=I_{D4}\times R_{ch4}=I_{D}\times R_{ch}$$

Now if we include the mismatches in Equation (29):

(30) $$I_{D}\times R_{ch}=\left(I_{D}+I_{off}+\Delta I_{D}\right)\left(R_{ch}+\Delta R_{ch}+R_{off}\right)$$

(31) $$\Rightarrow I_{D}\times R_{ch}=\left(I_{D}+I_{OS}\right)\left(R_{ch}+R_{OS}\right)$$

where $\left(\Delta R_{ch}+R_{off}\right)$ = $R_{OS}$, is the total equivalent offset resistance. From Equation (31) the term $\left(-\frac{I_{OS}}{I_{D}}\right)$ is given as:

(32) $$-\frac{I_{OS}}{I_{D}}=\frac{R_{OS}}{\left(R_{OS}+R_{ch}\right)}$$

Replacing Equation (32) in Equation (28):

$$V_{OSin}=\frac{1}{2}\sqrt{\frac{2I_{D}}{\mu_{p}C_{ox}\left(\frac{W}{L}\right)}}\left[\frac{\Delta (\frac{W}{L})}{\left(\frac{W}{L}\right)}+\frac{R_{OS}}{\left(R_{OS}+R_{ch}\right)}\right]+\left|\Delta V_{th}\right|$$

(33) $$\Rightarrow V_{OSin}=\frac{\left|V_{GS}-V_{th}\right|}{2}\left[\frac{\Delta (\frac{W}{L})}{\left(\frac{W}{L}\right)}+\frac{R_{OS}}{\left(R_{OS}+R_{ch}\right)}\right]+\left|\Delta V_{th}\right|.$$

From Equation (33), when the devices operate in the strong inversion region, the offset voltage largely depends on the overdrive voltage $\left|V_{GS}-V_{th}\right|$, mismatches in the device dimensions and the threshold voltage. Smaller tail current can be used to reduce the effect of the overdrive voltage on the offset voltage. Additionally, larger device dimensions will reduce the mismatches. Despite all these measures, the offset voltage cannot be entirely removed, a particular value of offset voltage will nevertheless persist in the design depending on the various process and temperature tolerances. In strong inversion region, a larger value of $I_{off}$ is required to have a significant effect on the offset voltage.

Weak inversion operation:

In the weak inversion region, the overdrive voltage or the mismatches in the device dimensions do not play any role for the offset voltage. For weak inversion region, the drain current varies exponentially with respect to the gate-source voltage. A much detailed explanation of the weak inversion operation is provided in [46,47]. The relationship in between the $I_{DSub-th}$ and $V_{GS}$ for a PMOS device is given by Equation (5) in Section 3.

From Equation (5), the expression for $V_{GS-Sub-th}$ in weak-inversion region can be given as:

(34) $$V_{GS-Sub-th}=nV_{T}\times \left[\ln\left(\frac{I_{D}}{I_{S}}\right)\right].$$

For the saturation or strong inversion region operation, the gate-source voltage depends on the $\frac{I_{D}}{g_{m}}$ ratio, but in case of weak inversion $g_{m}$ only depends on $I_{D}$. Moreover, for weak inversion $\frac{I_{D}}{g_{m}}\approx n\times V_{T}$ which is a constant. In practice, the value of *n* lies in between 1.5 to 1.6 and thermal voltage $V_{T}$ is 26mV for 300K, consequently $n\times V_{T}$ is a constant. Also, $I_{D}$ ≫ $I_{S}$, so by bringing a small variation in the drain current, $V_{GS}$ for both the transistor M3 and M4 will vary by a more substantial value.

By using the Relationship 34 in Equation (21), we get the expression for the offset voltage in the weak inversion region:

(35) $$V_{OSin}=nV_{T}\times \left[\ln\left(\frac{I_{D_{4}}}{I_{S}}\right)-\ln\left(\frac{I_{D_{3}}}{I_{S}}\right)\right].$$

(36) $$\Rightarrow V_{OSin}=nV_{T}\times \left[\ln\left(\frac{I_{D_{4}}}{I_{D3}}\right)\right].$$

Replacing $I_{D4}$ and $I_{D3}$ from Equations (16) and (18) respectively in Equation (36):

(37) $$V_{OSin}=nV_{T}\times \left[\ln\left(\frac{I_{D}}{I_{D}+I_{OS}}\right)\right]$$

Similar to the Equation (33), Equation (37) can also be expressed in terms of $R_{OS}$ and $R_{ch}$:

(38) $$V_{OSin}=nV_{T}\times \left[\ln\left(1+\frac{R_{off}}{R_{ch}}\right)\right]$$

If we compare both the equations of the offset voltage in strong and the weak inversion region following observations can be made :

- In weak inversion region, the variation in the drain current $I_{D}$ is the major contributor to the mismatches in absence of any $R_{off}$.
- The tolerances or spreading in the process factor and the device dimensions are the principal reason behind the offset voltage in the strong inversion region.
- The overall offset voltage is much smaller in the weak inversion region in comparison to the strong inversion one.
- In the weak inversion region, the offset voltage will largely depend on the variation created in the tail current of the differential pair due to $I_{off}$, which can be achieved by using a relatively small value of $I_{off}$.

**Figure 10:**
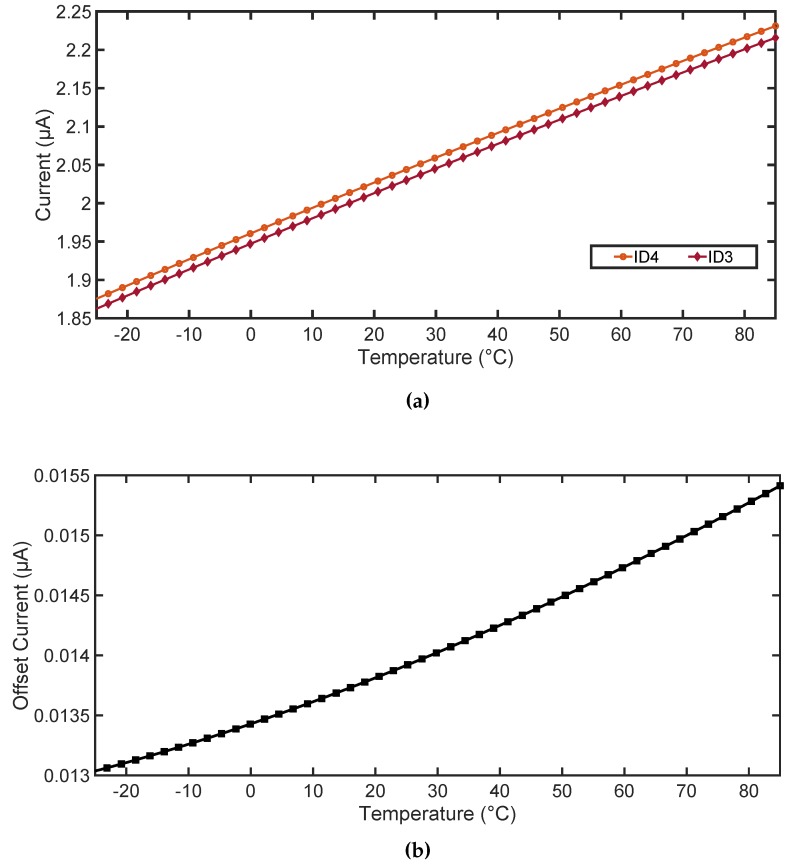
(**a**) Temperature dependence plot of the drain current $I_{D3}$ and $I_{D4}$ ; (**b**) Variation of Offset current $I_{off}$ due to temperature.

**Figure 11:**
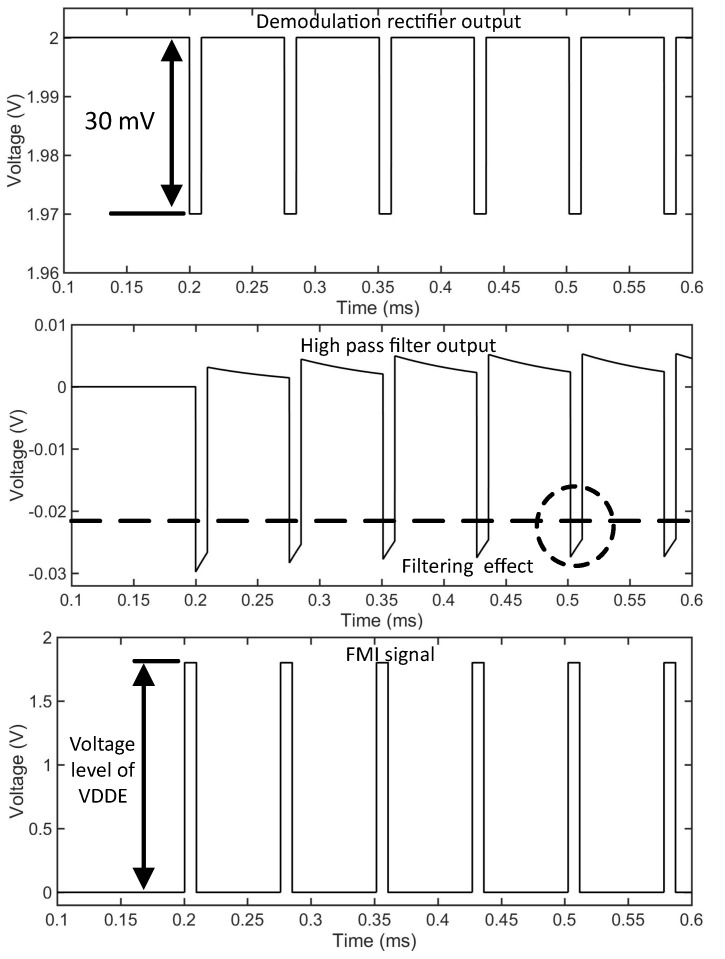
The output signals of the envelope detector, high pass filter, and the comparator (FMI).

Therefore, the comparator is designed to operate in the weak inversion region. The resistance $R_{off}$ results in small variation of the drain current, which introduces a preset offset in the circuit. To the best knowledge of the authors, this technique is unused for any other demodulator circuit. Figure 10 shows the temperature dependence of the drain currents $I_{D3}$ and $I_{D4}$ , consequently also the variation of the offset current $I_{off}$ with the temperature. At nominal temperature of 25 °C, $I_{off}$ is approximately 14fA. Figure 11 shows the simulated signals for envelope detector output, high pass filter output, and the demodulated signal FMI. The average value of $V_{off}$ ≈ 23mV ensures that the $V_{trig}$ lies much above VSS. The values obtained for $V_{OS}$ is within +15mV to +30mV for all possible mismatch conditions, as obtained from the simulation. The differential pair M3 and M4 along with M5 and M6 are kept in weak inversion region. The minimum designed slew rate for the circuit is 13V/µs and has a propagation delay of 293ns which is thirty times faster than the typical pulse width of the message signal. The power consumption of the comparator circuit is 16.1µW and has a layout area of $0.11\;mm^{2}$. A small hysteresis of 4mV is also provided using the resistors R1 and R2.

#### 4.2.3. Modulator

The communication from the tag to the reader happens via the load modulation scheme. The load modulation circuit discussed in [50] uses a conventional load modulation circuit, which includes a pair of resistor and a pair of active switching device connected to the antenna. Some of the practical problems regarding the load modulation in passive RFID tags, for different field strength conditions, are well explained in [68]. As mentioned in [68], in the presence of a strong field, the overvoltage protection circuit will clamp the voltage at the antenna including the modulation signal. This will result in a modulated signal too weak to be detected by the reader. According to the standard [66], the load modulation circuit shall be able to provide a minimum modulation depth of 10mV irrespective of the field strength.

**Figure 12:**
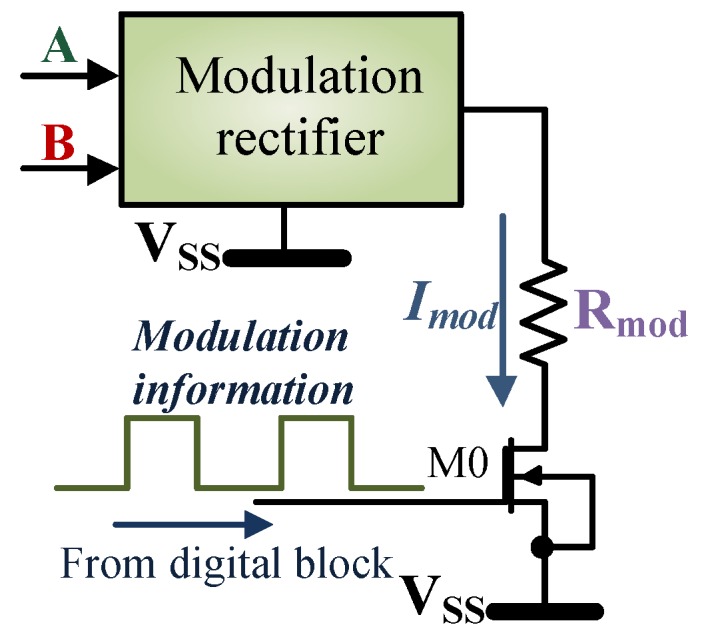
Schematic diagram of the load modulation scheme.

Figure 12 exhibits the load modulation circuit used for the design. The modulation rectifier ensures that there is no direct coupling between the modulation load $R_{mod}$ and the antenna so that the antenna tuning is not affected. The NMOS switch M0 is turned on by the FMO (Field Modulation Out) signal which is provided by the digital block and hence controls the modulation sequence. Each time the switch is turned on, it produces a current $I_{mod}$ = 3.6 to 4mA through $R_{mod}$ = 450Ω. This result in load impedance variation on the tag side which is detected by the reader. The modulation rectifier used is the same as the single power rectifier unit. In case the power rectifier unit itself is used, the modulation information will be damped out by the storage capacitor $C_{st}$. The source-drain metal width of the switch M0 is 5µm.

#### 4.2.4. Field Detector

**Figure 13:**
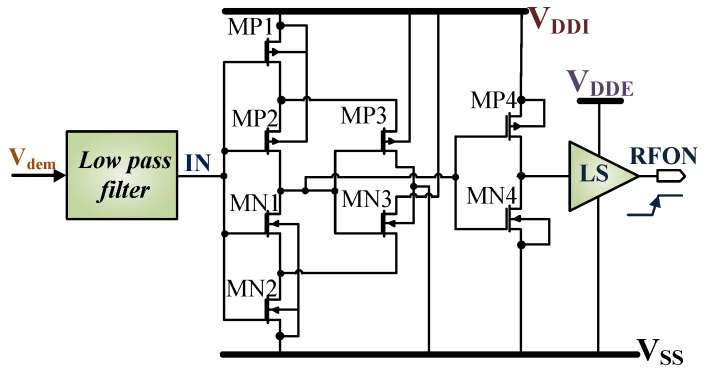
Field detector implemented using a Schmitt trigger circuit. LS indicates level shifter.

Figure 13 shows the Schmitt trigger circuit used for the field detection where the output RFON goes high when the field is detected at the input node IN. RFON is further utilized by the microcontroller as a reset logic. The low pass filter has a time constant of 42µs, which prevents RFON signal to alter its states instantaneously in case field is on or off for a limited period. When IN ≥ 1.3V, RFON transits from low to high and for IN ≤ 550mV it is low. It has a hysteresis of ≈560 mV for noise immunity. The switching on time for the circuit is ≈150 µs and switching off is ≈200 µs. It has a power consumption of 1.8µW.

#### 4.2.5. Clock Regenerator

**Figure 14:**
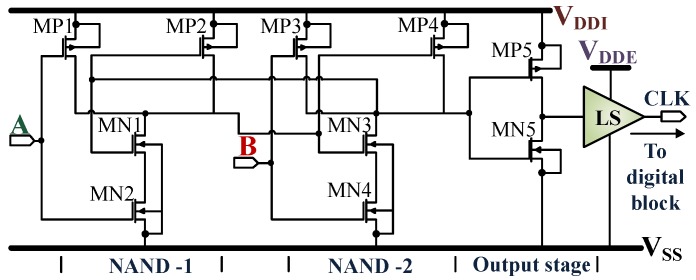
Clock regenerator extracts 13.56 MHz clocks (CLK) from the carrier signal using an RS flip-flop circuit. LS indicates level shifter.

An RS flip-flop circuit consisting of two NAND gates, an output stage, and a level shifter is used for the clock recovery as shown in Figure 14. The circuit extracts the 13.56MHz clock from the RFID/NFC field when the reader device is activated. The extracted clock pulse signal CLK has a designed duty cycle of 50±2%. The power consumption of the clock regenerator circuit is 3µW.

### 4.3. Summary of the Analog Block

**Figure 15:**
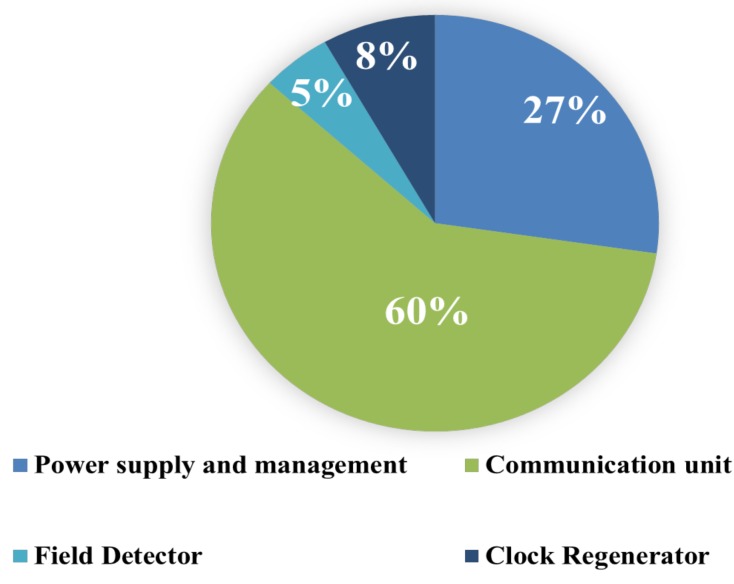
Power consumption distribution of the analog block obtained from the simulation.

When the reader device is active and $V_{dem}$ ≥ 1.7V, then $V_{REF}$ ≈ 1.21V (at 25°C). Next, $C_{st}$ is charged and $V_{DDI}$ ≈ 1.2V. When $V_{pow}$ ≥ $(V_{d2}$ + $V_{dS})$, $V_{DDE}$ is available. The CLK signal is activated and then the RFON signal. At this point, the IC is ready for the communication process. When the RF field is switched off, being a passive system, the entire module gets deactivated. Figure 15 shows the power consumption distribution of the analog block obtained from the simulation. The total power consumption of the analog block is 36µW where the communication unit consumes 21.2µW which is 60% of the total power consumption of the analog block. The power supply and management unit consume 9.8µW, Clock regenerator uses 2.9µW and, Field detector unit consumes 1.8µW.

## 5. Digital Block

**Figure 16:**
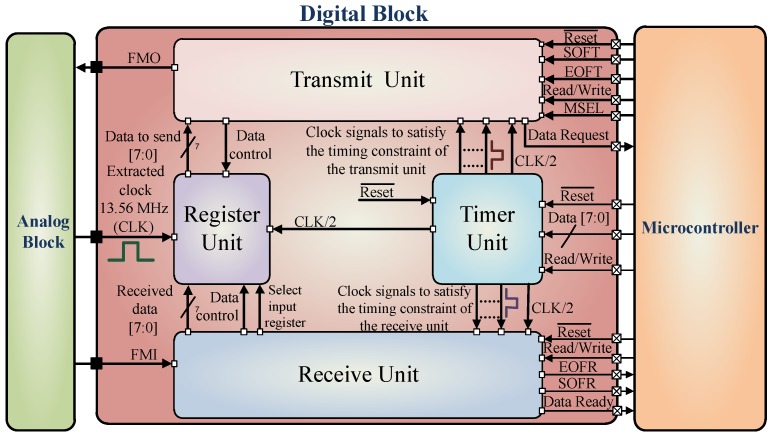
The design architecture of the digital block along with the interface signals.

Figure 16 shows the digital block of the IC together with all the internal modules. In between the digital and the analog block, there are three handshake signals—FMO, FMI, and CLK. The digital block extracts the message from the FMI signal and then prepares the required response together with the microcontroller, to be sent back to the reader by using the signal FMO. The digital block consists of mainly four functional blocks which are Transmit, Register, Timer and Receive unit.

The Timer-Unit generates the necessary clock signals from CLK required by the receive and the transmit unit for decoding and encoding messages respectively. The Register-Unit consists of a data register responsible for the entire data handling procedure. The data processing depends on whether the tag is in receiving (write mode) or transmitting (read mode). The Receive-Unit decodes the message sent by the reader and generates three signals: Data Ready, SOFR (start-of-frame-receive) and EOFR (end-of-frame-receive). These signals are further utilized by the microcontroller accompanied with the received data available from the 8-bit data bus - Data [7:0]. Each time a complete byte is received on the input stream, the ’Data Ready’ signal will go high which is further used by the microcontroller to drive an interrupt line. The received data is placed on the data bus to be further processed by the microcontroller. The ’EOFR’ and the ’SOFR’ signal goes high on receiving a valid EOF (end-of-frame) and SOF (start-of-frame) respectively.

The Transmit-Unit is responsible for transmitting the encoded message back to the reader by using the FMO signal. Each time the ’Data Request’ signal goes high (right after the previous byte is in the transmission process) it requests a new data byte on the data bus for further transmission and the signal is used by the microcontroller to drive an interrupt line. The transmission process is started by Setting the ’SOFT’ (start-of-frame-transmit) high, which starts off the ’SOF’ sequence and the first byte. Once the transmission sequence is started, the ’SOFT’ signal is ignored till the next ’Data Request’ signal. So far the frontend is in read-mode, the ’SOFT’ signal will be ignored. When ’EOFT’ (end-of-frame-transmit) signal is set to high, the transmission process is terminated. No further data request is generated instead an ’EOF’ sequence is generated to terminate the transmission process. The last byte in the transmission process will be send before the ’EOF’ sequence is generated.

**Figure 17:**
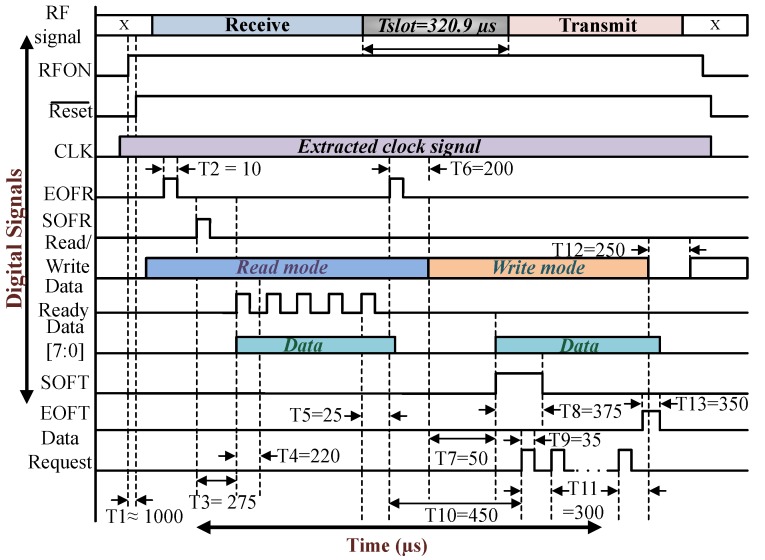
The timing diagram showing a typical receive and transmit operation.

The ’Read/Write’ signal controls the data direction, in case it is active high, data can be read from the bus (read mode). When the signal is low, the IC operates in write-mode, hence data can be written to the data bus. The ’MSEL’ is the modulation selector signal when set to active high it is in FM (frequency modulation) mode or (dual sub-carrier) mode. When set to active low it is in AM (Amplitude modulation) or (single sub-carrier) mode, which is also the default mode used. The ’$\bar{Reset}$’ is an active low signal used to reset the frontend. The detailed sequence of operation along with the corresponding timing constraints is shown in Figure 17. The timing sequences and the signals are generated as per requirement of the specification [69].

When the IC is in the read mode the ’Data Ready’ signal is set after each successful reception of the data byte. Whenever a new byte sequence is received, the earlier one is overwritten. This sequence is repeated until the complete byte stream is received, as shown in Figure 17. After the receive sequence is over there is a time delay before the transmit process starts which in turn is controlled by the timer unit in order to choose the slots to send back the response. In transmit mode the ’Read/Write’ signal goes to active low and the ’SOFT’ signal is set to active high before the next slot starts. This instant the first data byte is placed on the data bus. Each time a byte is transmitted successfully the data request signal requests the next byte as mentioned before. The number of data request pulse generated is dependent on the number of bytes to be sent. By setting the ’EOFT’ to active high the transmission process is terminated and an ’EOF’ signal is transmitted. The frontend is set back to receive mode and the signals ’SOFT’ and ’EOFT’ are set to active low.

## 6. Measurement Results and Discussion

**Figure 18:**
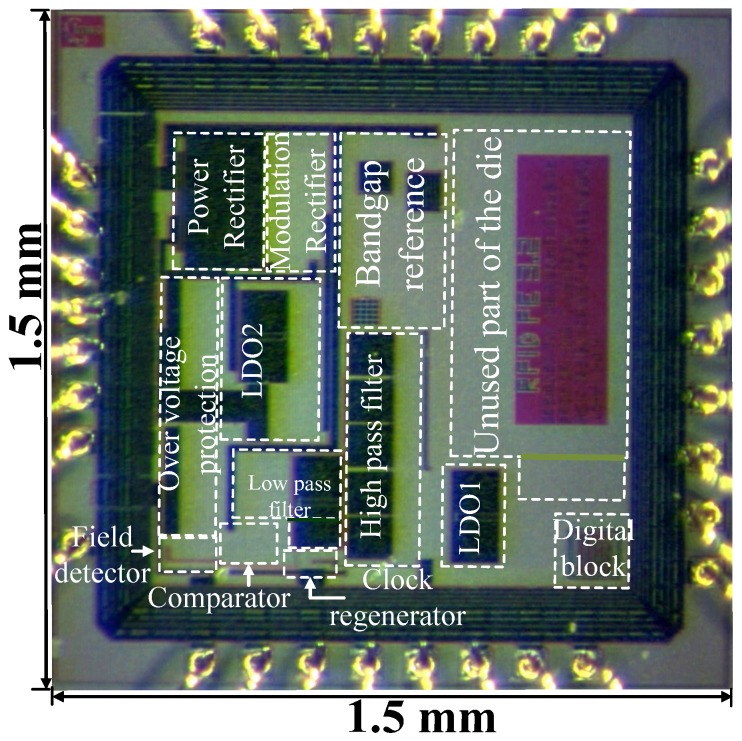
IC micro-photograph. Only top metal layer is visible.

The proposed frontend IC was fabricated in 0.18µm one-poly and six-metal CMOS technology with a total die area of 1.5mm×1.5mm and an effective area of $0.7\;mm^{2}$. Figure 18 shows the micro-photograph of the fabricated IC where nearly 40% of the die area is left unused. Additional circuitries like analog to digital converters and sensor interface circuits can be accommodated in the unused die area as mentioned in [65]. For a supply voltage of 1.2V, the digital core and input-output consumes 25µW and 46µW respectively, which is measured directly from the IC by using an external power source. The power consumption for the analog part is taken from the simulation as the part measurement of the analog power is not possible. Hence the total power consumption of the IC is ≈107 µW.

### 6.1. Power Supply and Management Block Measurements

**Figure 19:**
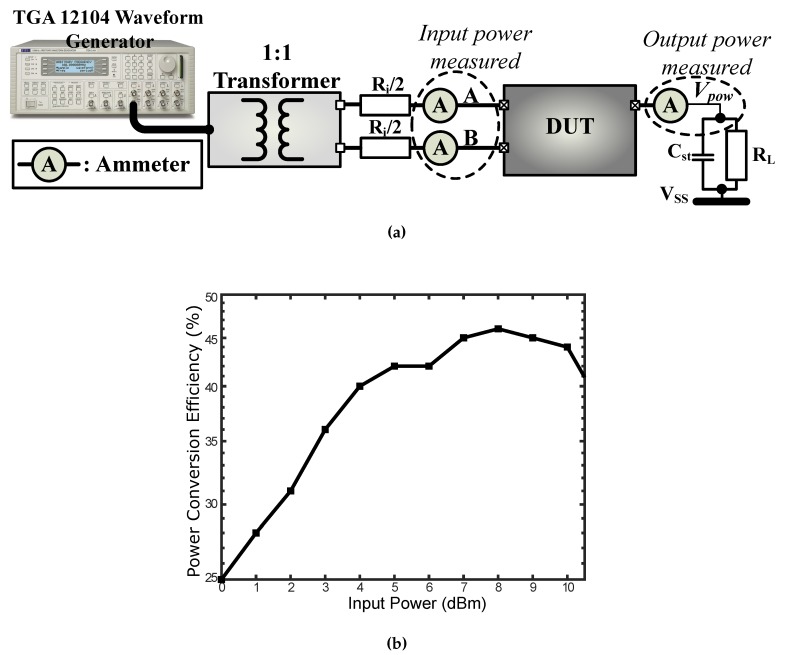
(**a**) A simplified view of the setup used for the measurement of the power conversion efficiency where DUT (device under test) is the frontend IC; (**b**) Measured power conversion efficiency of the power rectifier for an $R_{L}$ of 1kΩ.

Figure 19 shows the measured PCE (power conversion efficiency) for an $R_{L}$ (load) of 1kΩ, it can achieve a maximum PCE of 45%. The measurement setup includes a waveform generator (TGA 12104) with a source resistance of 50Ω which provides the 13.56MHz sinusoidal input signal. The output signal of the waveform generator is further connected to a 1:1 transformer with a solenoid core connected to a series resistance of $R_{i}$ = 440Ω.

### 6.2. Functional Test with a Passive Tag

**Figure 20:**
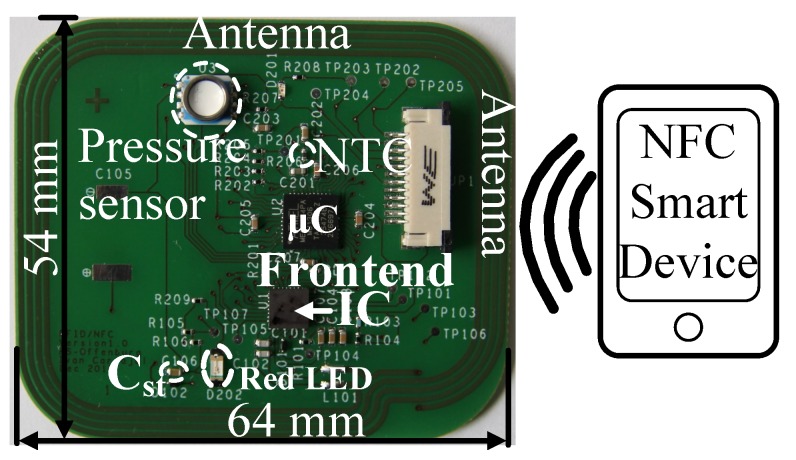
The passive tag used to realize the full functionality of the frontend IC. In the presence of the NFC field, the red LED is on.

Figure 20 shows the passive tag developed consisting of a µC (microcontroller) ATMEGA 164PA, a pressure sensor MS5803-30BA and a negative temperature coefficient (NTC) sensor. The tag antenna has an inductance of 3µH and a ceramic capacitor $C_{st}$ of 10µF is used for the temporary energy storage required for passive operation. For an NFC reader like Nexus 7 the effective read-range is 0.5 to 1cm depending on the orientation. The measured read-range achieved for an ISO15693 RFID reader (1W ± 1dB) is 14cm. At 14cm the modulation amplitude of the signal measured at the tag antenna sent by the reader is 50mV. In general, as the distance between the reader and tag increases the induced voltage at the tag antenna decreases. In reader to tag communication mode, with the increment in the distance, the modulation signal depth at the antenna input decreases. The demodulation circuit is able to detect a minimum modulation depth of 25mV, as ascertained from the measured FMI signal. Although the communication didn’t work beyond 14cm. Because in the tag to reader communication mode, as the distance between the reader and the tag increases, the modulation depth produced by the modulator circuit increases as the voltage input at the tag antenna decreases. This increment in the modulation depth affects the duty cycle of the clock signal produced by the clock regenerator circuit, which thereby results in distortion of the timing constraints required for a proper communication sequence. The passive tag can operate with a minimum field strength of 0.8A/m which is measured endowing with the standard [70] using a reference antenna and an 8pF, 10MΩ probe.

**Figure 21:**
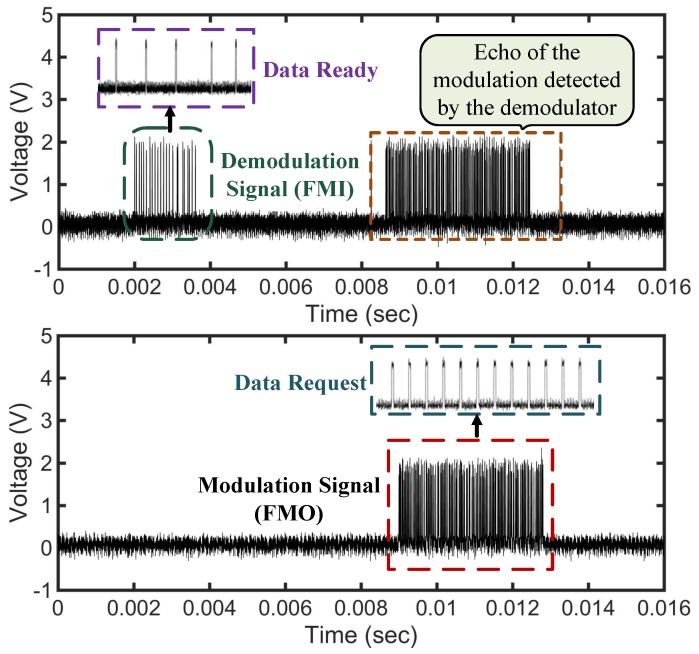
Measured demodulated, modulated, ’Data Ready’ and ’Data Request’ signal showing full communication process.

**Figure 22:**
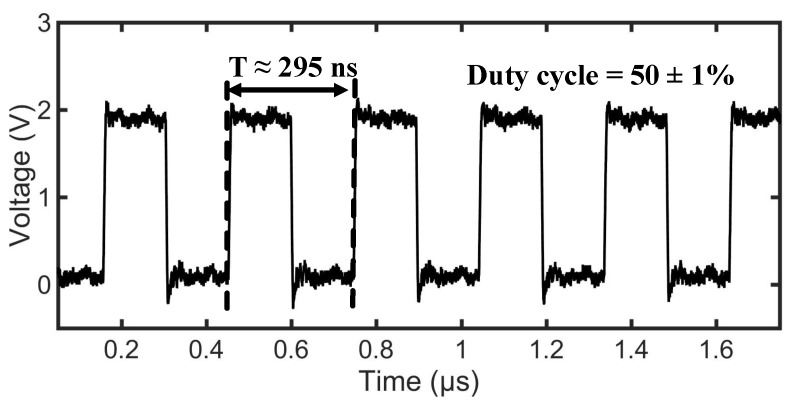
Clock extracted by the clock regenerator circuit has a frequency 13.56MHz which is further divided to $\frac{1}{4^{th}}$ of the frequency (3.39MHz) which is shown here.

**Figure 23:**
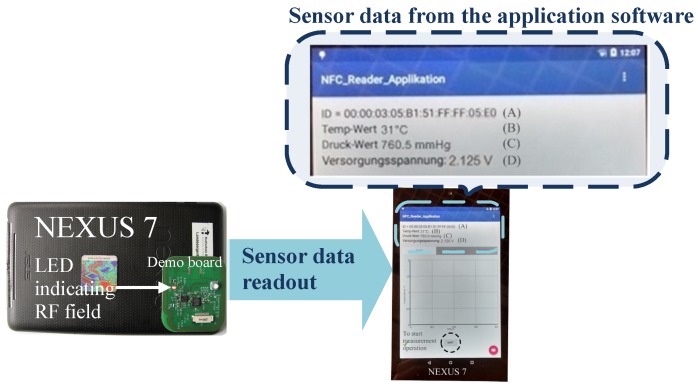
A typical measurement process involving the passive tag and an Android-based application software developed for the smart device.

Figure 21 depicts the entire communication process in between the reader and the tag. The communication signal sent to the reader is retrieved by the demodulator circuit denoted as FMI signal. The Data Ready signal indicates the 5 bytes of the command to get the UID (unique identification) of the tag. For a successful reception of a request, the microcontroller prepares the particular response and transmits it back to the reader using the FMO signal. The demodulator circuit detects the modulation signal as exhibited in Figure 21, but the digital logic of the IC ignores it as it is in the transmit mode. The Data Request signal shows the response prepared by the tag which is the 12 bytes of the tag UID. Figure 22 shows the FC4 clock extracted by the clock regenerator having a frequency of 3.39MHz and a duty cycle of 50±1%. Android-based application software is developed to readout the sensor data from the passive tag. Figure 23 shows the measurement process involving the passive tag and the smart device. In Table 3, the measured key parameters of the design are listed.

Table 4 conducts the comparison of this work with other state-of-the-art RFID or NFC ICs for different performance parameters. The contemporary works mentioned in Table 4 adhere to different standards of RFID or NFC hence they vary in terms of data rate. The authors propose here a FOM (figure-of-merit) defined as:

(39) $$FOM=\frac{1}{\left(Power\;Consumed\right)\times \left(Effective\;Die\;Area\right)}.$$

This work has a FOM of 9 which is better than the other contemporary design presented in Table 4. The effective use of sub-threshold operation and identifying system blocks which can be driven by a lower supply voltage of 1.2V helps to keep the power consumption low. The NFC frontend IC presented in [3], also yields a profound insight to the NFC system. The analog part of this work consumes 54% less power in comparison with the immediate contemporary design [3]. The works [4,71] also present state-of-the-art NFC systems, but they are not exactly comparable to this work, as they involve more complex circuit applications.

## 7. Conclusions

In this paper, a low power RFID/NFC frontend IC for passive tag applications is presented. The IC was fabricated in a 0.18µm–CMOS technology, having one poly and six metal layers. A novel approach is chosen to demodulate the ASK signal which consists of a comparator with a preset offset and an envelope detector circuit. The power consumption of the bandgap reference circuit is utilized as the load for the envelope detection. The RF transceiver and the digital interface are designed in conformance with the ISO/IEC 15693/NFC5 standard. Multiple power rails and weak inversion region device operation are used to keep the overall power consumption low. FOM proposed in this paper is applied for the comparison with the similar works. A passive tag using the frontend IC is developed to test the functionalities for full passive operation. Efficient design methods are employed, which aids to keep the power consumption low to 107µW. The power consumption of the analog part presented is 54% less than the recent state-of-the-art tag IC for a similar application. The IC has an effective die area of $0.7\;mm^{2}$, the remaining 40% empty die area can be used for additional circuitries like analog to digital converters and sensor interface circuits. The experimental results show the IC is capable of full passive operation and is suitable for inductively powered ultra-low-power biomedical or industrial sensor applications.

## Figures and Tables

**Table 1:** A brief overview of the available NFC standards.

	Type 1 [5]	Type 2 [6]	Type 3 [7]	Type 4 [8]	Type 5 [9]
Supported	ISO/IEC	ISO/IEC	JIS X 6319	ISO/IEC	ISO/IEC 15693
standard	14443 A	14443 A	–4 ( Felica)	14443 A/B	(18000-3)
Carrier			13.56 MHz		
Frequency			± 7 KHz		
Data rate	106kbps	106kbps	212/424kbps	106/212/	26.48kbps
				424kbps	
Modulation	ASK 100 %	ASK 100 %	ASK 10%	Standard A	10% or
(Reader to Tag)				+ ASK 10%	100% ASK
Data coding	modified	modified	Manchester	NRZ-L (Std B)	Pulse position
(Reader to Tag)	Miller	Miller	MSB first		mod. 1 out of
					256 / 1 out of 4
Modulation	Load	ASK 10%	Load	Standard A	Load mod.
	modulation (ASK)		modulation	+ Load mod.	
(Tag to Reader )	sub-carrier		with no	(BPSK) sub carrier	OOK/FSK
	(± 848kHz)		sub-carrier	(Std B)	sub-carrier
Data coding	Manchester	NRZ-L	Manchester	NRZ-L	Manchester
(Tag to Reader )					
Anti-collision	No	Yes	Yes	Yes	Yes

*Abbreviations:* ASK—Amplitude shift keying; BPSK—Binary phase shift keying; OOK—On-off shift keying; FSK—Frequency shift keying; NRZ-L—Non-return-to-zero level.

**Table 2:** Estimated values of $R_{i}$ for the corresponding effective peak voltages $\left(V_{e-pk}\right)$.

	$V_{e-\mathrm{pk}}\;\left(V\right)$	$R_{i}\;\left(\Omega \right)$	Operation Status
**a.**	<1	1000	No
**b.**	2.5	600	Yes
**c.**	3	440	Yes
**d.**	3.6	220	Yes
**e.**	4.4	180	No (voltage above safety level)

**Table 3:** Measured key parameters.

Parameter	Values
Carrier Frequency	13.56MHz ± 10kHz
Modulation type and index	ASK 10% NRZ
Data rate (max)	26.48kbps
Operating temperature	−30 °C to 85 °C
Bandgap reference voltage	1.21V ± 4mV
Demodulation depth (min)	25mV
Power consumption (analog)	36µW
Mode of operation	Passive
Technology	CMOS 0.18µm

**Table 4:** Comparison with state-of-the-art 13.56MHz-RFID and NFC frontend ICs for passive tag applications.

	This Work	[3]	[34]	[33]	[32]	[35]
CMOS process	0.18	0.18	0.18	0.18	0.35	0.35
(µm)						
Protocol	RFID ISO 15693	NFC	NFC	RFID ISO/	RFID ISO/	NFC
	/NFC 5			IEC 14443 (type-B)	IEC 18000	
Data rate	6.62 to 26.48	106 to 848	106 to 848	106	10 to 1000	106 to 212
kbps						
Power consumed	107	67.7 *	NA	360	960	NA
(µW)						
Die area	Effective 0.7	0.68	1.1	1.1	0.3182	7.92
(mm${}^{2}$)						
FOM	9	NA	NA	2.5	3.2	NA

NA indicates not available. * Only for analog block.
